# Identification and clinical impact of potentially actionable somatic oncogenic mutations in solid tumor samples

**DOI:** 10.1186/s12967-020-02273-4

**Published:** 2020-02-22

**Authors:** Sinead Toomey, Aoife Carr, Mateusz Janusz Mezynski, Yasir Elamin, Shereen Rafee, Mattia Cremona, Clare Morgan, Stephen Madden, Khairun I. Abdul-Jalil, Kathy Gately, Angela Farrelly, Elaine W. Kay, Susan Kennedy, Kenneth O’Byrne, Liam Grogan, Oscar Breathnach, Patrick G. Morris, Alexander J. Eustace, Joanna Fay, Robert Cummins, Anthony O’Grady, Roshni Kalachand, Norma O’Donovan, Fergal Kelleher, Aine O’Reilly, Mark Doherty, John Crown, Bryan T. Hennessy

**Affiliations:** 1 Medical Oncology Lab, Department of Molecular Medicine, Royal College of Surgeons in Ireland, RCSI Smurfit Building, Beaumont Hospital, Dublin, Ireland; 2 Department of Medical Oncology, St. James’s Hospital Dublin, Dublin, Ireland; 3 Data Science Centre, Royal College of Surgeons in Ireland, Dublin, Ireland; 4 Department of Pathology, Royal College of Surgeons in Ireland, Dublin, Ireland; 5 Department of Pathology, St. Vincent’s University Hospital, Dublin, Ireland; 6 Department of Pathology, Royal Victoria Eye and Ear Hospital, Dublin, Ireland; 7 Princess Alexandra Hospital, Brisbane, Australia; 8 Department of Medical Oncology, Beaumont Hospital, Dublin, Ireland; 9 National Institute for Cellular Biotechnology, Dublin City University, Dublin, Ireland; 10 Department of Medical Oncology, St. Vincent’s University Hospital, Dublin, Ireland

## Abstract

**Background:**

An increasing number of anti-cancer therapeutic agents target specific mutant proteins that are expressed by many different tumor types. Successful use of these therapies is dependent on the presence or absence of somatic mutations within the patient’s tumor that can confer clinical efficacy or drug resistance.

**Methods:**

The aim of our study was to determine the type, frequency, overlap and functional proteomic effects of potentially targetable recurrent somatic hotspot mutations in 47 cancer-related genes in multiple disease sites that could be potential therapeutic targets using currently available agents or agents in clinical development.

**Results:**

Using MassArray technology, of the 1300 patient tumors analysed 571 (43.9%) had at least one somatic mutation. Mutations were identified in 30 different genes. *KRAS* (16.5%), *PIK3CA* (13.6%) and *BRAF* (3.8%) were the most frequently mutated genes. Prostate (10.8%) had the lowest number of somatic mutations identified, while no mutations were identified in sarcoma. Ocular melanoma (90.6%), endometrial (72.4%) and colorectal (66.4%) tumors had the highest number of mutations. We noted high concordance between mutations in different parts of the tumor (94%) and matched primary and metastatic samples (90%). *KRAS* and *BRAF* mutations were mutually exclusive. Mutation co-occurrence involved mainly *PIK3CA* and *PTPN11*, and *PTPN11* and *APC*. Reverse Phase Protein Array (RPPA) analysis demonstrated that PI3K and MAPK signalling pathways were more altered in tumors with mutations compared to wild type tumors.

**Conclusions:**

Hotspot mutational profiling is a sensitive, high-throughput approach for identifying mutations of clinical relevance to molecular based therapeutics for treatment of cancer, and could potentially be of use in identifying novel opportunities for genotype-driven clinical trials.

## Background

Traditionally most cancers were categorised in terms of their tissue of origin, the size and nodal status of the primary tumor, and the presence of metastatic lesions. For solid tumors, the origin of the tumor was generally the deciding factor in assessing treatment options, and patients were usually assigned to different treatment options based on primary tumor diagnosis or site, histological subtype, nodal status, hormone receptor and HER2 status for breast and gastro-oesophageal cancers, and *KRAS* and *EGFR* status for colorectal and lung cancers, respectively.

With the discovery that many tumors contain mutations within oncogenes or tumor suppressor genes that may predict responses to targeted anti-cancer therapies, genomic profiling to support treatment decisions is used in some settings. Well established examples include *KIT* mutations which are present in ~ 85% of gastro-intestinal stromal tumors [1], *EGFR* mutations that have been identified in ~ 15% of non-small cell lung cancers [2], and lung and colorectal cancers with mutations in the *KRAS* oncogene [3]. Several agents have been developed to target these molecules including the tyrosine kinase inhibitor imatinib, which induces clinical responses in gastrointestinal stromal tumors that harbour *KIT* mutations [4], and erlotinib and gefitinib which are effective in non-small cell lung cancers with mutations or insertions/deletions in *EGFR* [2, 5]. *BRAF* V600E mutations are found in approximately half of all cutaneous melanomas, and the use of BRAF inhibitors in these patients has been shown to improve survival [6–8]. In metastatic colorectal cancers the use of EGFR inhibitors in combination with conventional chemotherapy significantly improved survival [9, 10]. Furthermore, afatinib and osimertinib have recently been approved for the treatment of EGFR mutated non-small cell lung cancers [11, 12], while the combination of BRAF and MEK inhibitors improve overall survival in melanoma [13]. However, the presence of mutations in proteins other than the intended therapeutic target can affect the response to a particular therapy. For example, lung and colorectal cancers with mutations in *KRAS* or *BRAF* do not respond to treatment with anti-EGFR therapies [3].

Oncogene mutations do not usually occur randomly, but are more frequent in certain genomic regions [14]. Because genomic aberrations can predict responsiveness to targeted therapies, profiling cancer mutations will allow a greater understanding of the pathways involved in driving the cancers growth, and ultimately allow for the genetic and/or molecular characteristics of the tumor to play a role in determining the choice of therapy. This process will maximise the efficacy of treatment while minimising undesirable side effects resulting from altered drug metabolism due to the patient’s genetic background. Genomically guided therapies may be of particular use in treating rare tumors, where very large randomised trials are often impractical [15]. Currently most genomic technologies to profile samples for the clinical selection of patients for targeted therapies assess the mutational status of one or a few genes (e.g. pyrosequencing) or investigate a specific histologic phenotype [immunohistochemistry and fluorescence in situ hybridisation (FISH)]. These approaches can miss multiple alterations that are also potentially targetable, and other alterations that may be markers of resistance to standard therapies. While next generation sequencing (NGS) has made it possible to test multiple genes simultaneously, tumor molecular profiling by NGS remains challenging in the clinical setting. Whole genome or exome sequencing is not feasible for many clinical labs due to the large amount of data required to detect low level variants and the time and bioinformatics expertise needed to analyse the data.

In the present study, we have used MassArray technology, a high-throughput mass spectrometry-based technique which enables sensitive and rapid somatic mutation profiling in solid tumor samples. The MassArray technology makes it possible to analyse multiple hotspot mutations within 3 days, and negates the need for complex bioinformatic analysis. Initial studies using this technology showed it to be highly advantageous for somatic mutation profiling. A study by Thomas et al. used a somatic mutation panel, comprising of 238 oncogene mutations in 17 oncogenes, to screen 1000 human tumor samples from 17 types of solid tumors. Relevant mutations were confirmed in 30% of the samples, and novel mutations were detected that had not been previously reported due to the sensitivity of this method [16]. Since then numerous other studies have demonstrated the feasibility of using MassArray to identify actionable mutations for the purpose of implementing genome-driven oncology programs [17–20], and the technology has recently been approved as a clinical diagnostic platform [18].

Emerging evidence suggests that many of the genomic aberrations currently used to guide the selection of targeted therapies within specific disease contexts may also occur in other cancer types. The identification and targeting of these biomarkers provides an opportunity to extend the benefits of personalised medicine to a larger population of patients. The aim of this study was to determine the type, frequency, overlap and functional proteomic effects of potentially targetable recurrent somatic hotspot mutations in 47 cancer-related genes in multiple disease sites that could be potential therapeutic targets using currently available agents or agents in clinical development.

## Methods

### Tumor samples

Formalin-fixed paraffin embedded (FFPE) tumor samples were obtained from tumor banks under the auspices of Institutional Review Board-approved protocols at Beaumont Hospital Dublin, Ireland (613 samples), St. Vincent’s University Hospital, incorporating the Royal Victoria Eye and Ear Hospital, Dublin, Ireland (470 samples), and St. James’s Hospital, Dublin, Ireland (217 samples). Tumor selection was based on the most prevalent cancers treated in our hospitals and the availability of sufficient tissue from the tumor. In total, the tumors of 1300 patients were evaluated. More than one region of the tumor was evaluated from 50 patients, and a matched metastatic sample was evaluated from 30 patients. All patients were diagnosed between January 1994 and October 2014.

### Sample preparation

In each case, up to 6 × 10 µM sections were cut from the paraffin block. Sandwich hematoxylin and eosin staining was performed on the first and last sections and checked by an experienced pathologist to ensure that tumor was present throughout the sections. If the tumor content was lower than 50%, tumor area was macrodissected. DNA was extracted using an All Prep DNA FFPE kit (Qiagen) as per manufacturer’s instructions. DNA concentration was calculated using the Qubit ds DNA Kit. A total of 10 ng DNA from each sample was subjected to beta-globin gene (300 base pairs) polymerase chain reaction and analysed on a 1.5% Agarose gel to verify DNA quality. Only DNA samples with successful β-globin amplification were subjected to further analysis.

### Mutation analysis

Mass-spectrometry-based single nucleotide polymorphism genotyping technology (Agena Biosciences, Hamburg, Germany) was used for identification of hotspot, potentially clinically relevant nonsynonymous somatic mutations (Additional file 1: Table S1). Because these mutations were known to be somatic from previous studies, no germline samples were included in this analysis. The genes were further subdivided by pathway, and include MAPK, PI3K and related pathway genes (Additional file 1: Table S2). Assays were designed using strict assay design parameters optimized for sensitive mutation detection. The panel consisted of 31 multiplex assays capable of detecting 504 somatic hotspot mutations in 47 genes. Ten nanograms (10 ng) of DNA was added to each PCR reaction and DNA was amplified using custom designed PCR primer pools. Unincorporated nucleotides were inactivated using shrimp alkaline phosphatase, and a single base extension reaction was performed using extension primers that hybridise immediately adjacent to the mutations of interest. Salts were removed by adding a cation-exchange resin, before the multiplexed reactions were spotted onto SpectroCHIP II arrays. Matrix chips were analysed on an Agena MassArray MALDI-TOF system.

### Protein extraction and RPPA analysis of tumors

From each tumor, up to 5 × 10 µM sections were cut from the paraffin block. Protein extraction was carried out as previously described [21] using a 20-mM Tris buffer pH 9 containing 2% SDS and protease inhibitors (Roche Applied Science Cat. # 04693116001 and 04906845001) lysis buffer.

RPPA analysis was carried out as previously described by us [22, 23]. The antibodies used are in supplementary data (Additional file 2: Table S3). The data was normalised by protein loading using the entire antibody panel.

### Statistical analysis

Mutation calls for each sample were determined using visual inspection and Typer Software based on mass spectra. Reactions where > 15% of the resultant mass ran in the mutant sites were scored as positive. Overall survival (OS) was measured in months from the date of diagnosis to the date of death from any cause, and progression free survival (PFS) from the date of diagnosis to the date of documented radiological recurrence or death from any cause in the absence of documented recurrence. The Kaplan–Meier method and the log rank test were used to analyse the associations between survival [progression free survival (PFS) and overall survival (OS)] for groups of interest. The statistical significance of co-occurring mutations was determined using Fisher’s exact test. Differences of p < 0.05 were considered statistically significant. Statistical analysis was performed with GraphPad Prism version 5.01.

## Results

### Sample set and clinicopathologic characteristics

Archival paraffin embedded tumor tissue from 1300 cancer patients was analysed. Patient characteristics are detailed in Table 1 and Additional file 1: Figure S1. Five hundred and ninety three patients (45.6%) were male and 707 (54.4%) were female. The median age was 63 years (range 19–90). Thirty nine patients had metastatic disease at the time of diagnosis. The tumor types were colorectal (n = 354, 27.2%), lung (n = 223, 17.2%), breast (n = 172, 13.2%), prostate (n = 83, 6.4%), melanoma (n = 65, 5%), lymphoma (n = 49, 3.8%), gastric (n = 45, 3.5%), head and neck (n = 45, 3.5%), bladder (n = 38, 2.9%), ocular melanoma (n = 32, 2.5%), endometrial (n = 29, 2.2%), kidney (n = 28, 2.2%), ovary (n = 24, 1.8%), brain (n = 24, 1.8%), oesophagus (n = 22, 1.7%), pancreas (n = 19, 1.5%), liver (n = 16, 1.2%), testis (n = 16, 1.2%), thyroid (n = 13, 1%) and sarcoma (n = 3, 0.2%). Two samples were tested in 80 patients. Of these, 50 patients had two regions of the primary tumor tested, while 30 patients had a primary and a metastatic site analysed. Patients who had more than one sample tested were considered mutated for a specific gene if mutations were seen in any of the samples tested.

**Table 1:** Classification of the samples studied by age and clinical characteristics (n = 1300)

Clinical characteristic	Number of patients (%)
Gender	
Male	593 (45.6)
Female	707 (54.4%)
Age at diagnosis	
Median (range)	63 (19–90)
Tumor type	
Colorectal	354 (27.2%)
Lung	223 (17.2%)
Breast	172 (13.2%)
Prostate	83 (6.4%)
Melanoma	65 (5%)
Lymphoma	49 (3.8%)
Gastric	45 (3.5%)
Head and neck	45 (3.5%)
Bladder	38 (2.9%)
Ocular melanoma	32 (2.5%)
Endometrial	29 (2.2%)
Kidney	28 (2.2%)
Ovary	24 (1.8%)
Brain	24 (1.8%)
Oesophagus	22 (1.7%)
Pancreas	19 (1.5%)
Liver	16 (1.2%)
Testis	16 (1.2%)
Thyroid	13 (1%)
Sarcoma	3 (0.2%)
Origin of the tumors	
Primary tumor	1166 (89.7%)
Metastasis	44 (3.4%)
Unknown	90 (6.9%)

### Mutation detection

**Fig. 1:**
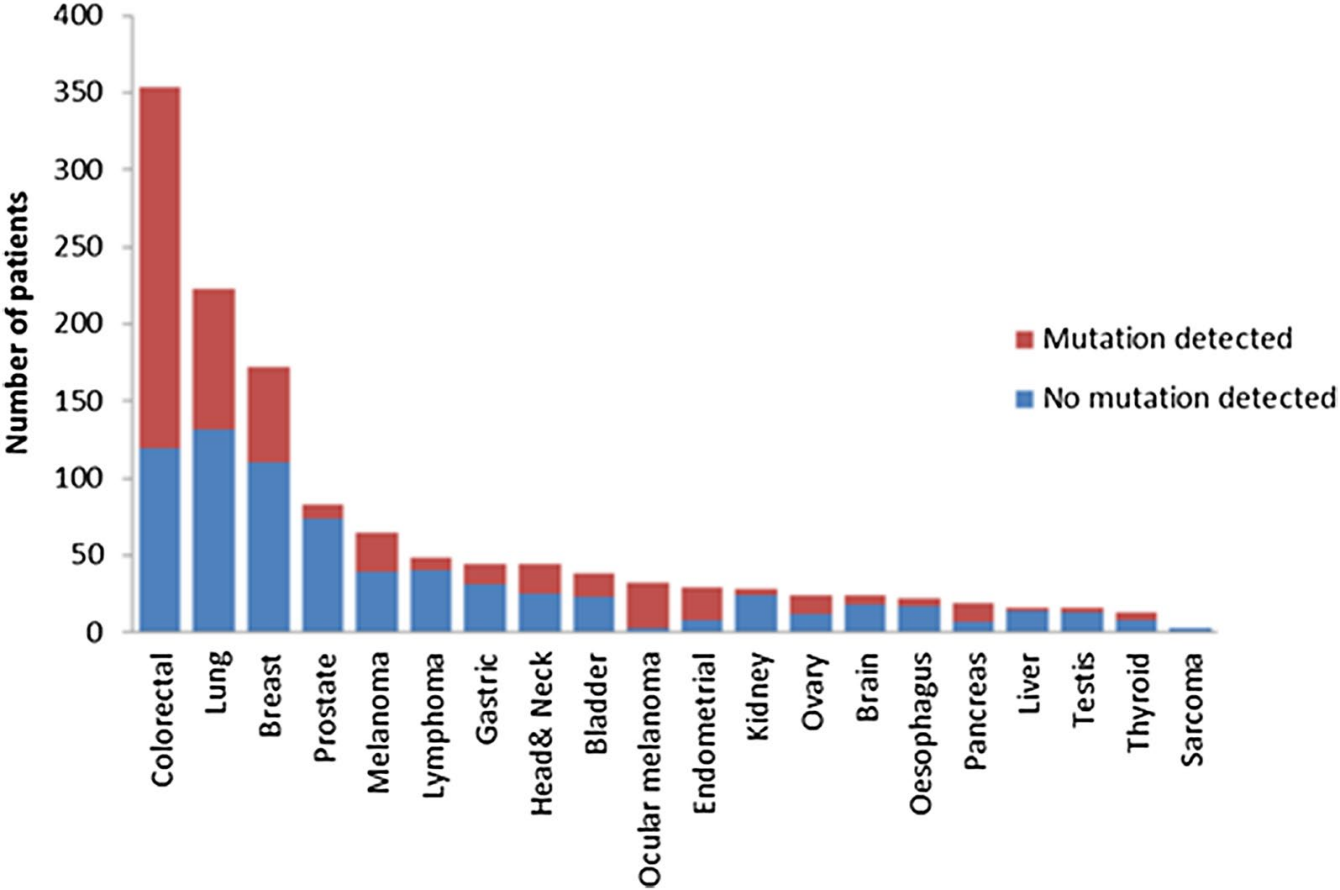
Number of patients with somatic mutations according to tumor type. The most common tumor type profiled was colorectal, followed by lung, breast, prostate and melanoma. Mutations were most frequently identified in ocular melanoma (90.6%), endometrial (75.4%), and colorectal (66.4%) tumors

Genotyping was performed for 504 somatic hotspot mutations in 47 genes. Among the 1300 patient tumors tested, 571 patients (43.9%) had at least one potentially targetable alteration. A gene was considered targetable if there is either an approved or investigational therapy targeting the product of the gene either directly or indirectly, although this may not reflect current availability or therapeutic efficacy in different tumor types. Proportionately ocular melanoma had the greatest number of potentially targetable mutations (90.6%, 29/32), followed by endometrial (72.4%, 21/29) and colorectal (66.4%, 235/354) cancers, while liver (12.5%, 2/16), prostate cancer (10.8%, 9/83) and sarcoma (0% 0/3) had the lowest number of potentially targetable mutations (Fig. 1).

**Fig. 2:**
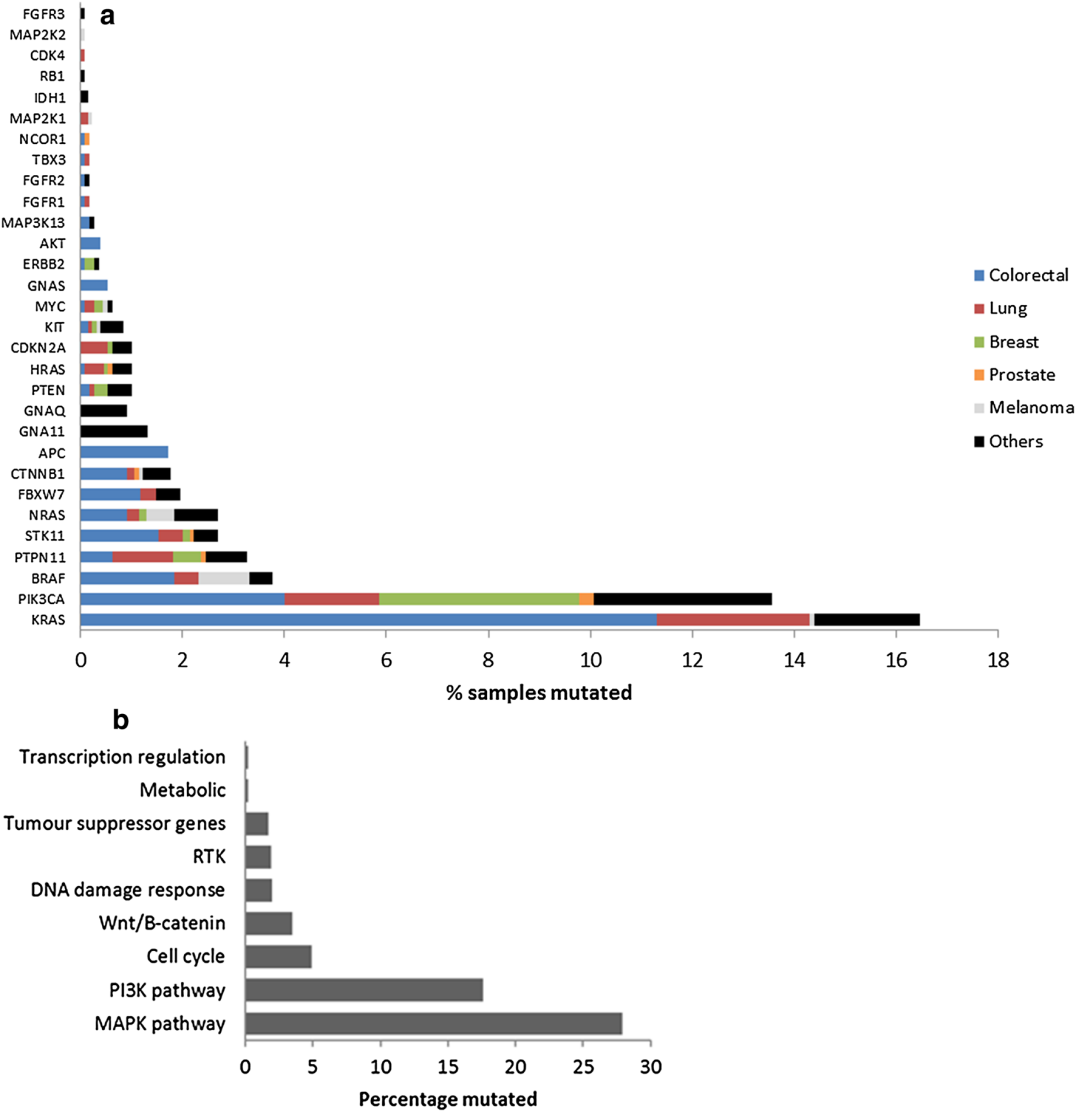
**a** Frequency of genomic mutations across human tumor types. Other tumors include lymphoma, gastric, head and neck, bladder, ocular melanoma, endometrial, kidney, ovary, brain, oesophagus, pancreas, liver, testis, thyroid and sarcoma. **b** Frequency of genomic mutations in human tumor samples by pathway

The most frequently mutated genes were *KRAS* (16.5%) and *PIK3CA* (13.6%). Somatic alterations were also detected in *BRAF* (3.8%), *PTPN11* (3.3%), *STK11* (2.7%), *NRAS* (2.7%), *FBXW7* (2%), *CTNNB1* (1.8%), *APC* (1.7%), *GNA11* (1.3%), *PTEN* (1%), *HRAS* (1%), *CDKN2A* (1%), *GNAQ* (0.9%), *KIT* (0.9%), *MYC* (0.6%), *GNAS* (0.5%), *ERBB2* (0.4%), *AKT* (0.4%), *MAP3K13* (0.3%), *FGFR1* (0.2%), *FGFR2* (0.2%), *TBX3* (0.2%), *NCOR1* (0.2%), *MAP2K1* (0.2%), *IDH1* (0.2%), *RB1* (0.1%), *CDK4* (0.1%), *MAP2K2* (0.1%) and *FGFR3* (0.1%) (Fig. 2a). Mutations in *KRAS* were most frequently identified in colorectal cancers, followed by lung cancers. Mutations in *PIK3CA* were most frequently identified in breast cancers and lung cancers, while *BRAF* mutations were most frequently identified in colorectal cancers and melanomas. Full details of the percentage of mutations found in each tumor type are shown in Fig. 2a and Additional file 3: Table S5. When the somatic mutations were subdivided by pathway, 27.9% of tumors had a MAPK pathway mutation, while 17.6% of tumors had a PI3K pathway mutation (Fig. 2b). Details of the genes included in each pathway are shown in Additional file 1: Table S2. Analysis using the Cancer Genome Interpreter demonstrated that 126/132 (95%) of mutations identified were known or predicted tumour drivers and 123/126 (98%) of these were Tier 1 mutations, which are known to change the activity of the gene product in a way that promotes oncogenic transformation. Results of Cancer Genome Interpreter analysis are shown in Additional file 2: Table S3.

We also investigated the mutational status of two regions of the primary tumor from 50 patients and matched primary and metastatic tumors from 30 patients. Mutations were similar in the different regions from the primary tumor, and also in matched primary and metastatic tumors. Samples taken from two different regions of the same tumor had an overall concordance rate of 94% (47/50) (Additional file 1: Table S6), indicating a degree of tumor heterogeneity in these samples. Matched primary and metastatic tumors demonstrated an overall concordance rate of 90% (27/30) (Additional file 1: Table S7). One breast cancer patient had a *PTPN11* mutation in the primary tumor that was not identified in the metastatic tumor, another breast cancer patient had an *ERBB2* mutation in the metastatic tumor that was not identified in the primary tumor, and one lung cancer patient had a *MAP3K13* mutation in the metastatic tumor that was not identified in the primary tumor (Additional file 1: Table S7). The absence of these mutations was verified by droplet digital PCR, an ultrasensitive mutation detection technology.

### Co-occurrence and mutual exclusivity

**Fig. 3:**
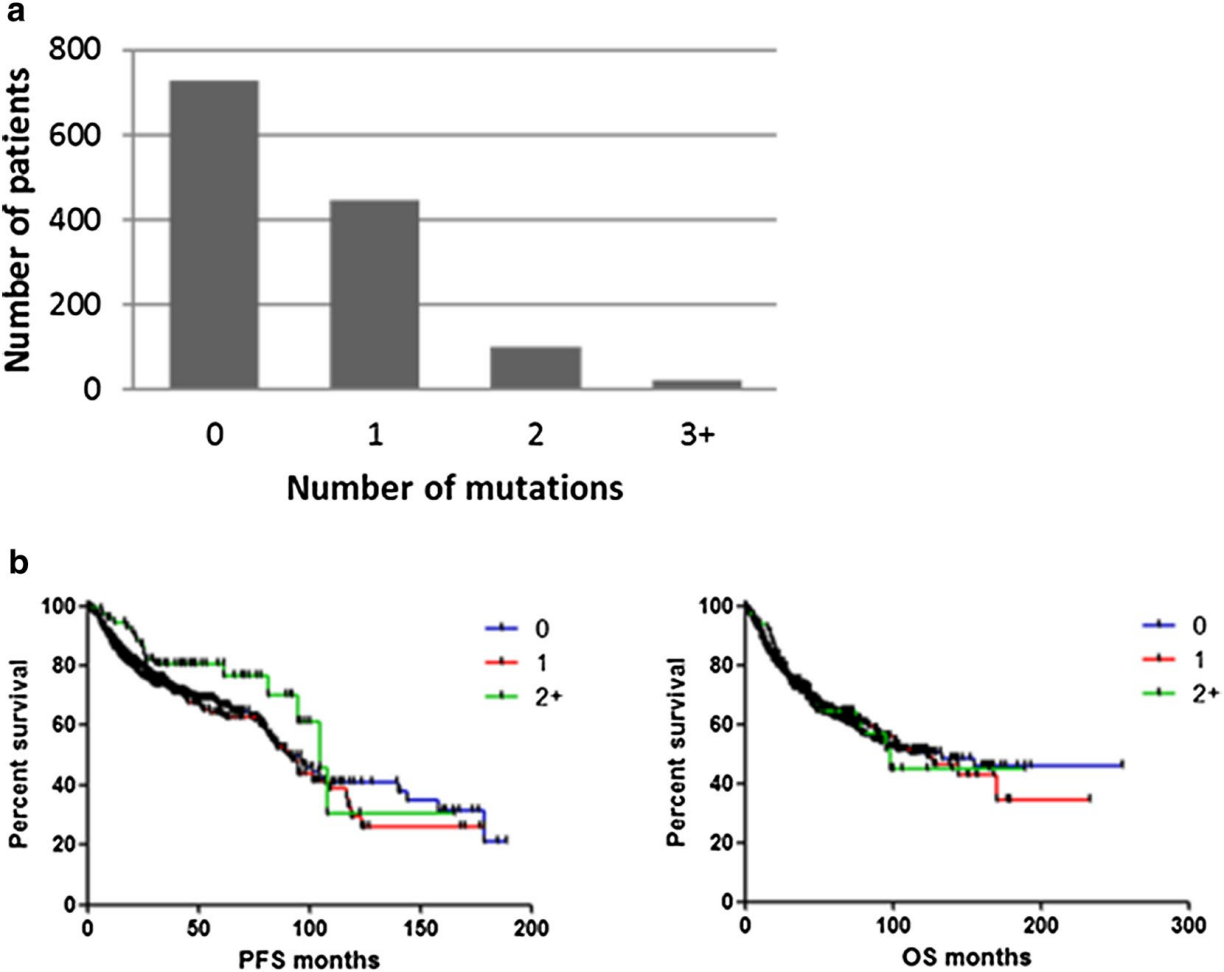
**a** Number of genomic mutations per patient tumor. **b** Correlation between number of mutations and patient survival

Of the 1300 patients analysed, 446 (34.3%) had a single targetable somatic mutation in their tumors. *KRAS* was the most common single mutation, and was found in 146 (32.7%) of these patients. This was followed by *PIK3CA* mutations, which were present in 114 (25.6%) patients. 101 (7.8%) patients had two targetable mutations in their tumors, while 24 (1.8%) patients had 3 or more targetable mutations in their tumors (Fig. 3a). There was no statistically significant difference in progression free survival (PFS) or overall survival (OS) in patients with no mutations or patients with one or two or more targetable mutations in their tumors (Fig. 3b).

**Fig. 4:**
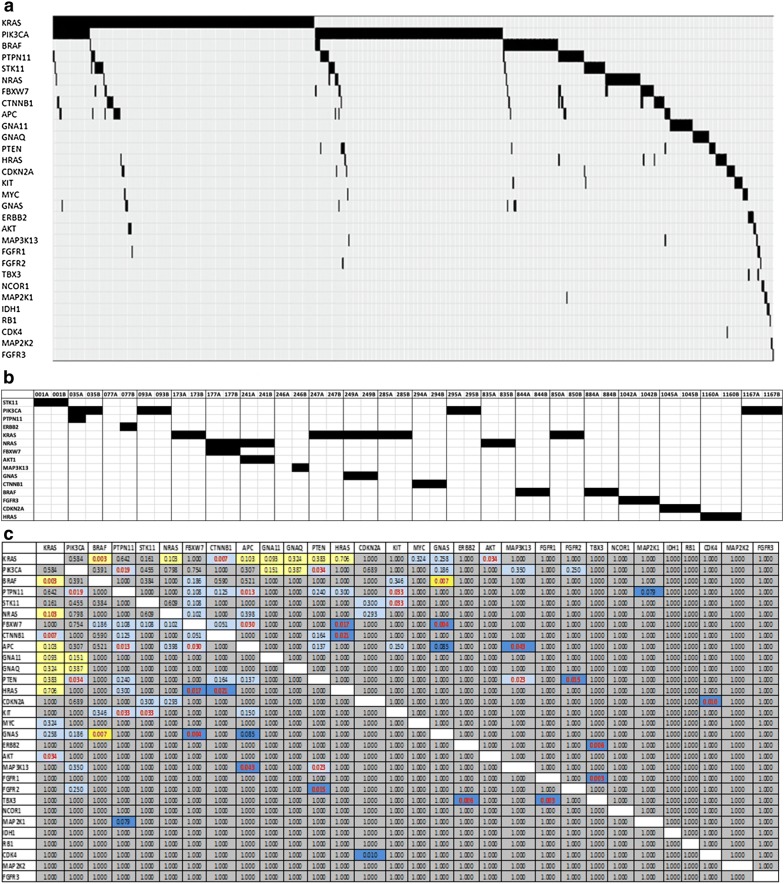
**a** Mutually exclusive and co-occurring oncogene mutations in human tumors. Mutations were grouped together when the occurred within a given gene. **b** Mutation co-occurrence in primary and metastatic pairs. **c** Incidence of co-occurring mutations. Grey indicates no association of mutations (0.5 odds ratio < 2), Pale yellow indicates some tendency toward mutual exclusivity (0.1 < odds ratio < 0.50), dark yellow indicates strong tendency toward mutual exclusivity (0 < odds ratio < 0.1), light blue indicates tendency toward co-occurrence (2 < odds ratio < 10), dark blue indicates strong tendency toward co-occurrence (odds ratio > 10)

We also examined the co-occurrence and mutual exclusivity of mutations found in our cohort (Fig. 4a). 78.1% of tumors with mutations (446/571) had only 1 targetable mutation identified, while 21.9% (125/571) had co-existing mutations. We noted mutual exclusivity between *KRAS* and *BRAF* mutations (p = 0.003), and the co-occurrence of somatic mutations in *PIK3CA* and *PTPN11* (p = 0.019), *PIK3CA* and *PTEN* (p = 0.034), *PTPN11* and *APC* (p = 0.013), and *PTPN11* and *KIT* (p = 0.033). We also noted the co-occurrence of mutations in *STK11* and *KIT* (p = 0.033), *FBXW7* and *APC* (p = 0.030), *FBXW7* and *HRAS* (p = 0.017), *KRAS* and *CTNNB1* (p = 0.007) and *HRAS* and *CTNNB1* (p = 0.021) (Fig. 4b). *APC* and *KRAS* mutations co-occurred in 10/152 (6.6%) of colorectal tumours, with a tendency towards mutual exclusivity. However, it should be noted that only a limited number of *APC* mutations were analysed in our study. Somatic mutations in PIK3CA and KRAS frequently co-occurred in colorectal, lung, head and neck, gastric and endometrial tumors (Additional file 1: Table S8).

### Correlation of frequent mutations with patient outcomes

**Fig. 5:**
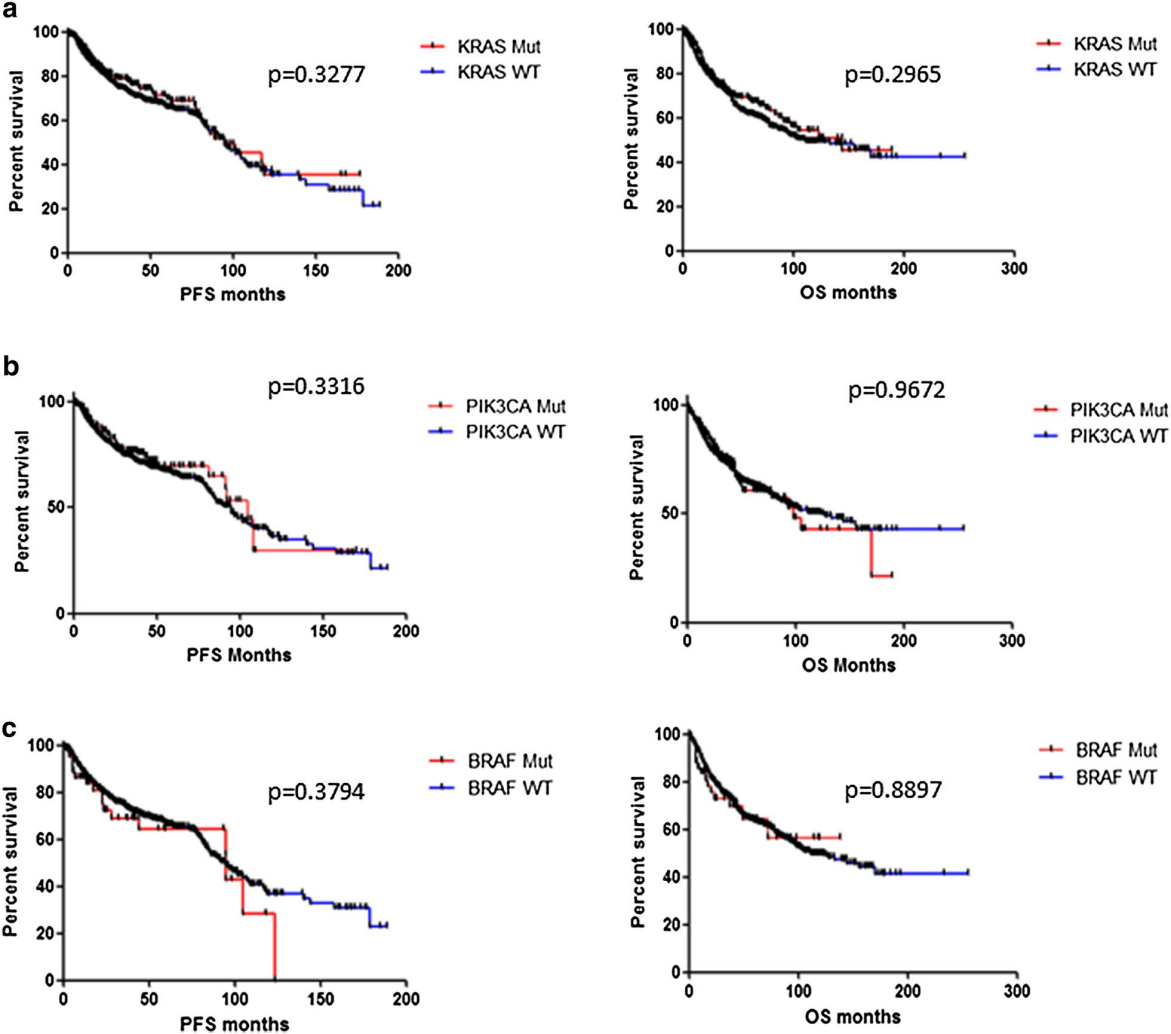
Correlations between somatic mutation status and patient survival. No significant differences were found in progression free survival (PFS) and overall survival (OS) between patients with **a** KRAS wild-type tumors and KRAS mutated tumors; **b** PIK3CA wild-type and PIK3CA mutated tumors; **c** BRAF wild-type and BRAF mutated tumors

**Fig. 6:**
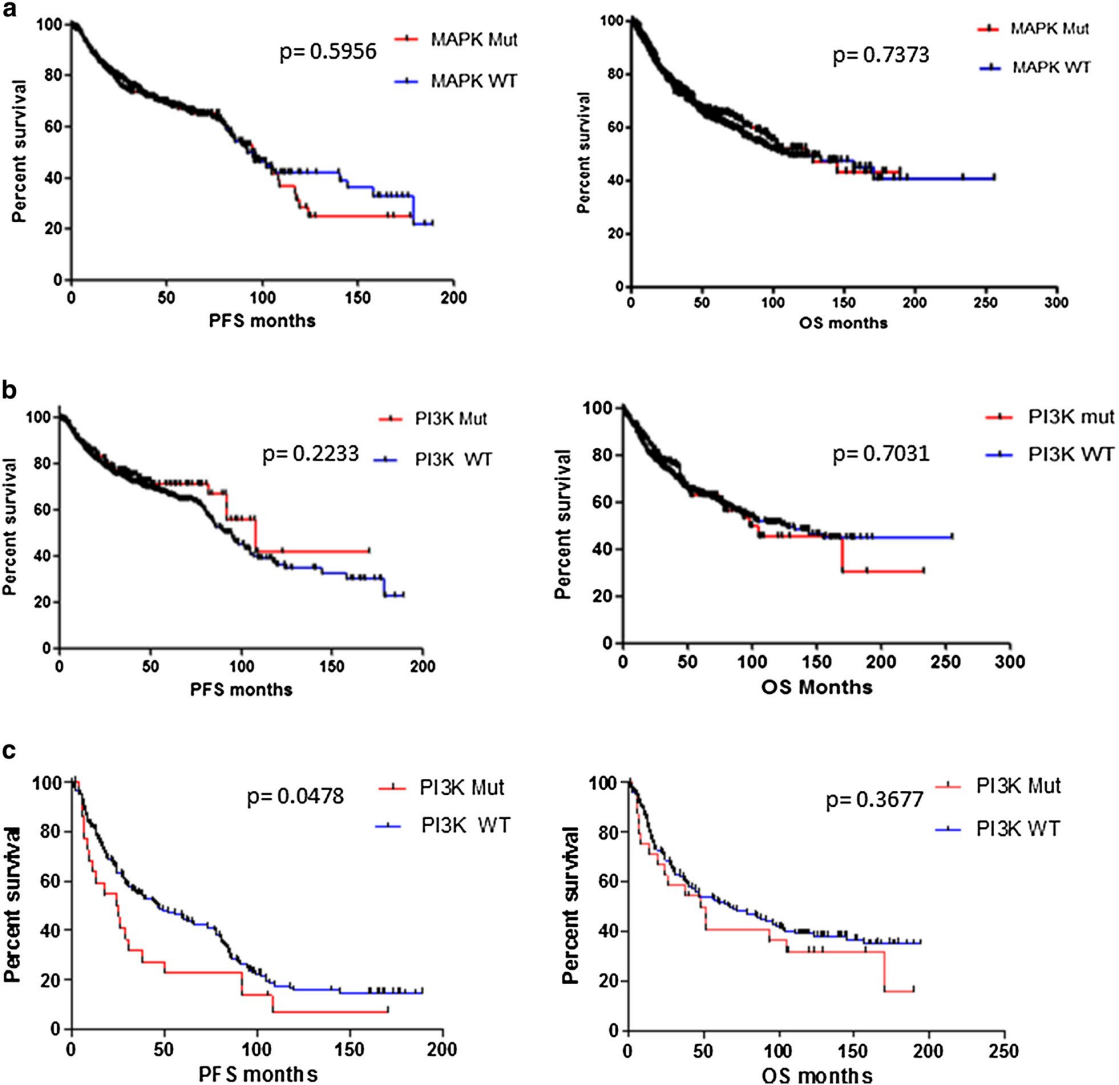
Correlations between pathway somatic mutation status and patient survival. No significant differences were found in progression free survival (PFS) and overall survival (OS) between patients with **a** MAPK pathway wild-type tumors and MAPK pathway mutated tumors; **b** PI3K pathway wild type and PI3K pathway mutated tumors. **c** Lung cancer patients with PIK3CA pathway mutated tumors had significantly poorer PFS than lung cancer patients with PI3K pathway wild-type tumors (24.95 months vs. 46 months, p = 0.0478)

*KRAS, PIK3CA* and *BRAF* were the three most frequently occurring somatic gene mutations in our cohort. There was no statistically significant difference observed in median PFS or OS between patients with a *KRAS* mutation in their tumors, and those with no *KRAS* mutation in their tumors. Similarly the presence of either a *PIK3CA* or a *BRAF* mutation had no statistically significant impact on PFS or OS overall (Fig. 5). When mutations were subdivided by pathway, there was no significant difference observed in median PFS in patients with mutations in the *MAPK* pathway (p = 0.5956) or in patients with mutations in the *PI3K* pathway (p = 0.2233) compared to patients with tumors wild type for mutations in the corresponding pathway. There was also no difference in overall survival (Fig. 6). However, lung cancer patients whose tumors harboured PI3K pathway mutations had significantly shorter PFS than lung cancer patients with PI3K pathway wild type tumors (24.95 months vs. 46 months, p = 0.047) (Fig. 6).

### Effect of aberrations in the PI3K, MAPK and related pathways on PI3K and MAPK pathway activation

**Fig. 7:**
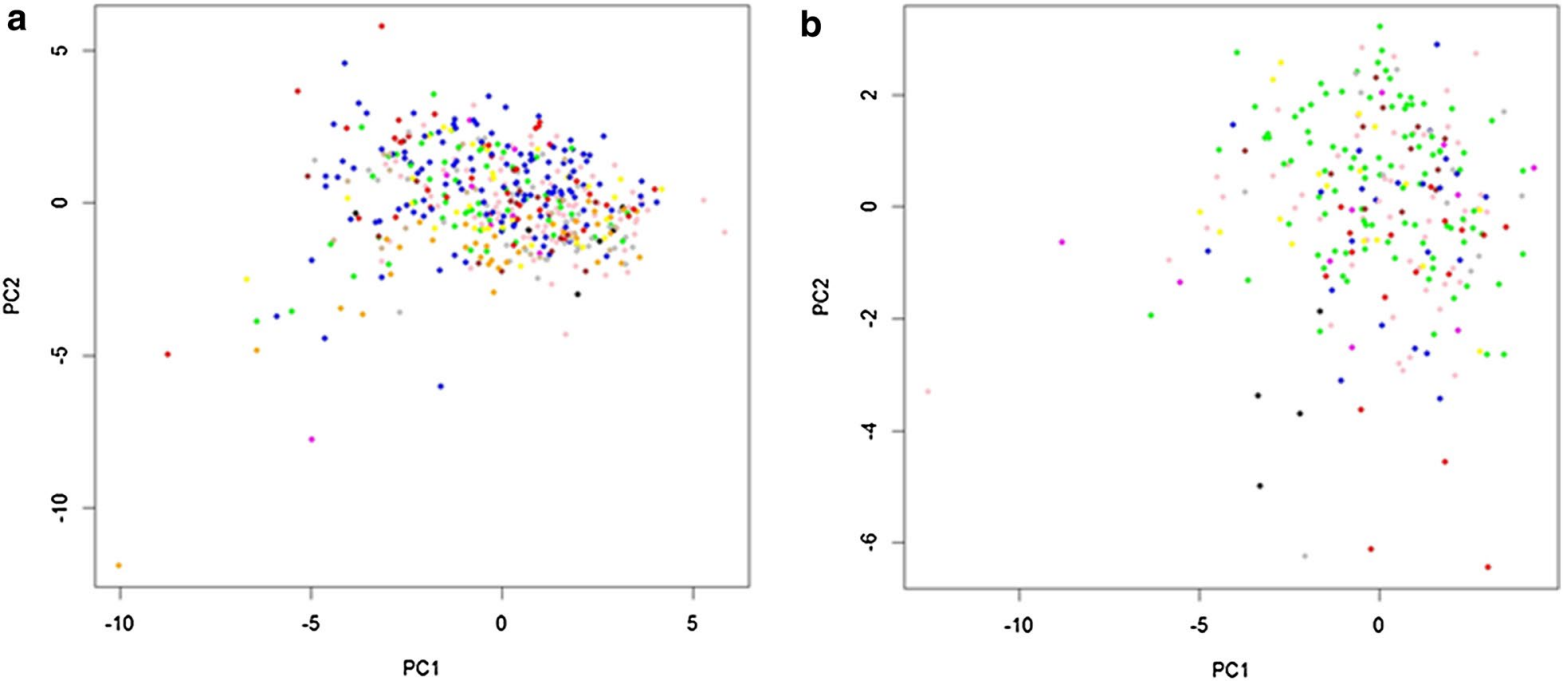
Principal component analysis (PCA) of RPPA data. **a** Principal components 1 and 2 for all wild type tumors and **b** principal components 1 and 2 for mutated tumors. Blue = bladder, kidney and prostate cancer; Red = breast cancer; Green = colorectal cancer; Grey = cervical, endometrial and ovarian cancer; Black = gastric and oesophageal cancer; Yellow = melanoma and ocular melanoma; Magenta = head and neck cancer; Brown = liver cancer; Orange = lung cancer; tan = lymphoma; Pink = testicular cancer

We applied RPPA to determine if genomic mutations in the PI3K, MAPK and related signalling pathway genes activated the PI3K and MAPK pathways. PCA analysis demonstrated no clear difference in pathway activation between mutated and wild type tumors, however proteins were more consistently expressed in the wild type tumors. In contrast, in the mutated tumors, protein expression was more variable, suggesting that there were more proteins activated or repressed in the mutated tumors (Fig. 7).

## Discussion

Next generation sequencing technologies are still not fully cost effective for most clinical laboratories, and hotspot mutational profiling can be accomplished in a more acceptable timeframe for use in routine clinical care. Furthermore, hotspot mutations are more likely to be driver mutations and less likely to be incidental genetic variants [24, 25]. In this study, we have used MassArray analysis to analyse the type and frequency of potentially targetable hotspot somatic mutations in a heterogenous population of solid tumor patients. Activating mutations in the PI3K and MAPK signalling pathways occur frequently in many cancers and have been implicated in the development of resistance to chemotherapy and radiotherapy [26–29]. Therefore, we investigated the frequency of mutations in key gene components of these and related signalling pathways (504 somatic mutations in 47 genes).

Mutations in 30 different genes were identified in 571/1300 (43.9%) of patients in our study. *KRAS* and *PIK3CA* were the most frequently mutated genes, and were identified in 16.5% and 13.6% of patients, respectively. There are conflicting reports in the literature about the prognostic value of *PIK3CA* mutations in solid tumors. While a number of studies have suggested that the presence of a *PIK3CA* mutation confers a poor prognosis in several cancers including breast, lung and colorectal [30–32], other studies have reported no significant difference between patients harbouring *PIK3CA* mutations and those with wildtype *PIK3CA* in their tumors [33–35], which is consistent with our findings. However, there are currently several ongoing trials targeting *PIK3CA* mutated tumors with PI3K inhibitors, with some promising results. In the Solar-1 trial in hormone receptor positive, HER2 negative advanced breast cancer patients, patients with *PIK3CA* mutated tumors who received the PI3K inhibitor alpelisib in combination with fulvestrant had significantly improved progression free survival compared to patients who received fulvestrant alone (11 months vs. 5.7 months; p = 0.00065) [36]. Furthermore, other PI3K inhibitors have also demonstrated anti-tumor responses in patients with advanced solid tumors and non-Hodgkin lymphomas [37, 38].

The frequency of *KRAS* mutations in our cohort was 16.5%, similar to findings reported in the MSK IMPACT Clinical Sequencing Cohort (16%) [39]. *KRAS* has long been considered “undruggable” and for many years has remained an elusive target for drug therapy. However, we have considered *KRAS* to be potentially targetable, as there are currently several ongoing trials targeting the MAPK pathway in *KRAS* mutant patients [40]. A recent study of AMG-510, a small molecule that specifically and irreversibly inhibits *KRAS* G12C, demonstrated anti-tumor activity when administered as monotherapy to patients with *KRAS* G12C mutated advanced solid tumors [41].

In our overall cohort, we did not find any high frequency recurrent mutation to be associated with PFS or OS. Other studies have shown that pathway level alterations rather than recurrent single gene mutations predict response to therapy [42]. Although we did not find alterations in the PI3K or MAPK pathway to be associated with PFS or OS in our overall cohort, we did find that lung cancer patients with somatic PI3K pathway mutations in their tumors had significantly shorter median PFS than patients with PI3K pathway wild type tumors. However, it is important to note that although we found no difference between mutated and non-mutated tumors in terms of OS, < 5% of our cohort were treated with molecularly targeted therapies.

Colorectal and lung tumors were the two most representative tumor types in our cohort with 354 and 223 patients, respectively. Mutations were identified in 66.4% of colorectal tumors with *KRAS* (41.5%) and *PIK3CA* (14.7%) being among the most frequently mutated genes, in line with previous studies [43]. The frequency of *APC* mutations, which occur at a high frequency in colorectal cancer, in our cohort was lower than previously observed (12.4%). Although mutations tend to occur within a small part of the APC gene (codons 1286–1513) there are few hotspot mutations [44]. Therefore, our hotspot mutation panel may not be the most appropriate method for identifying *APC* mutations. In the lung tumor cohort, mutations were most frequently identified in *KRAS* (17.5%), *PIK3CA* (10.8%), *PTPN11* (5.4%) and *CDKN2A* (3.1%). Almost half (48.4%) of our lung cancer patients had lung squamous cell carcinoma (SCC), which is often perceived to lack molecular targets, however our results suggest that *KRAS*, *PIK3CA*, *PTPN11* and *CDKN2A*, in particular, could potentially be actionable targets in lung SCC.

One hundred and twenty five (9.6%) patients in our cohort had co-occurring mutations and interestingly, even using a targeted panel with a limited number of genes, 24 (1.8%) patients had 3 or more genes mutated. Mutation co-occurrence occurred frequently with *PIK3CA* and *KRAS* genes in lung, colorectal, head and neck, gastric and endometrial tumors. Previous studies have also shown that mutations in *PIK3CA* are not mutually exclusive with *KRAS* mutations and the two are known to commonly co-exist [45, 46], confirming the parallel activation of the PI3K and MAPK pathways in the tumors of many patients in our cohort.

Our study also utilised paired samples from two different regions of the same tumor, and from primary and metastatic sites from the same patient, allowing us to determine the concordance of mutations in these samples. We found a high concordance rate between two different regions of the same tumor (94%) and between the temporally distant recurrent/metastatic site (90%), suggesting that these mutations are homogenous within the tumor, and agreeing with previous studies that oncogenic drivers are typically shared by all sites of disease, even in patients with heavily pretreated and advanced cancers. Our results suggest that archival tissue as well as newly biopsied samples may be suitable for initial genomic profiling in many patients; however, it is important to note that none of these patients had received targeted therapy. Following targeted therapy, genomic evolution and selection pressure often lead to mediators of acquired resistance becoming the dominant clone as demonstrated by the emergence of the *EGFR* T790M mutation in *EGFR* mutated non-small cell lung cancer patients treated with first or second generation tyrosine kinase inhibitors [47]. In this setting liquid biopsies may be more appropriate for molecular stratification of tumors to guide targeted therapy selection.

Currently several ongoing clinical trials are assessing the utility of genomically informed personalised cancer therapy. The NCI-MATCH trial has almost 40 treatment arms investigating targeted therapies against specific genomic aberrations. Some promising results have emerged from these studies so far. The FGFR kinase inhibitor AZD4547 showed some activity, with acceptable toxicities, in various solid tumors harbouring *FGFR* fusions [48]. Furthermore, the AKT inhibitor capivasertib reduced tumor size in 23% of patients with *AKT1* gene mutations who received the drug. A further 46% of patients had stable disease [49]. The prospective phase II EXACT trial demonstrated longer PFS when individualised treatment regimens were used and suggested that treatment based on real-time molecular profiling leads to superior clinical benefit [50]. The I-PREDICT study takes into account several actionable molecular alterations in the tumor, with the hypothesis that targeting only one molecular alteration in a tumor is likely to be insufficient to produce a durable anti-tumor response, to propose drug combinations for patients. In this study, 23% of patients who received matched therapy with nonstandard drug combinations, which overall were well tolerated, experienced an objective response [51]. Recently larotrectinib, a pan-TRK inhibitor, and entrectinib, a pan-TRK, ALK, and ROS1 tyrosine kinase inhibitor, were the first tumor-agnostic-targeted therapies developed and approved in oncology for the treatment of pediatric and adult tumors that have an *NTRK* gene fusion [52].

Functional proteomic analysis using RPPA showed that although the PI3K and MAPK signalling pathways are more altered in mutated tumors than in wild type, all of the tumors have alterations in these signalling pathways regardless of the mutation status of the tumor or the type of mutation. This suggests that even in the absence of a specific genomic mutation, these patients could potentially benefit from treatment with a multitargeted tyrosine kinase inhibitor (TKI). There are several multitargeted drugs already used in the treatment of various cancer types [53]. One such drug, regorafenib, improves PFS and OS in patients with refractory colorectal cancer independent of tumor mutation status [54].

We are aware that our study has several limitations. Only hotspot mutations in specific genes of interest were evaluated, making the method unsuitable for genes like *APC*, which are highly mutated but have few hotspots. Secondly, because the majority of our samples were primary tumors only a minority of patients received targeted therapies in this setting, making it impossible to determine the impact of targetable somatic mutations on response to targeted therapy. The number of paired primary/metastatic samples in our study was limited, and although we observed high concordance between the primary tumor and recurrence/metastatic site, these were all first recurrences and concordance is likely to be less at later recurrence, particularly in patients who have received targeted therapies. Furthermore, our study does not examine epigenetic and transcriptomic changes involved in cancer pathogenesis. Disruption of key epigenetic regulators by mutation leads to an altered transcriptome, which can multiply the effect of a single genetic alteration [55]. Therefore, complete understanding of transcriptomic and epigenomic changes in the tumor could compliment the molecular information obtained from hotspot mutation profiling, as described herein.

## Conclusions

Although the clinical utility of genomic profiling of tumors has not been fully demonstrated, cancer treatment is moving to a new paradigm where the molecular characteristics of the tumor are used to inform treatment decisions. Larotrectinib, entrectinib and pembrolizumab have been approved across cancer types based solely on the mutational status of the tumor [56, 57]. The present study aimed to identify key alterations that may represent important targets for novel therapies. Although not without its limitations, using a hotspot mutation profiling approach avoids complex NGS designs and bioinformatics analysis, can be accomplished in a realistic timeframe for use in day-to-day clinical care, and could be used to expand treatment options for patients with cancer.

## Supplementary information

**Additional file 1: Figure S1.** Breakdown of tumour types in A. Colorectal B. Lung and C. Breast tumour cohorts. **Table S1.** List of mutations analysed using the Agena MassArray technology. **Table S2.** Somatic mutations tested subdivided by pathway. **Table S4.** Antibodies used for Reverse Phase Protein Array (RPPA), including the company from which it was purchased, the catalog number, the host species and the dilution at which it was used. **Table S6.** Somatic mutation status in samples taken from two different regions of the same primary tumour. Samples identified in one sample but not in the other are identified in bold print. **Table S7.** Somatic mutation status of primary and matched metastatic tumour samples. Samples identified in one sample but not in the other are identified in bold print. **Table S8.** Frequency and co-occurrence of somatic PIK3CA and KRAS mutations in solid tumour samples.

**Additional file 2: Table S3.** Cancer Genome Interpreter mutation analysis of the mutations identified in solid tumour samples analysed in our study.

**Additional file 3: Table S5.** Percentage of somatic hotspot mutations in each tumour type.

## Data Availability

All data generated or analyzed during this study are included in this published article. Raw and processed data are stored in the laboratory of BH and are available upon request.

## Abbreviations

DNA: Deoxyribonucleic Acid
FFPE: Formalin Fixed Paraffin Embedded
FISH: Fluorescence in situ hybridisation
MAPK: Mitogen-activated protein kinase
NGS: Next generation sequencing
OS: Overall survival
PCA: Principal component analysis
PFS: Progression free survival
PI3K: Phosphoinositide 3-kinase
RPPA: Reverse Phase Protein Array
TKI: Tyrosine kinase inhibitor
