# Supplementary material for: Identification and clinical impact of potentially actionable somatic oncogenic mutations in solid tumor samples
Source: J Transl Med. 2020 Feb 22;18:99. doi: 10.1186/s12967-020-02273-4 (PMC7036178; doi:10.1186/s12967-020-02273-4)
Supplement: Supplementary file 1 — Additional file 1: Figure S1. Breakdown of tumour types in A. Colorectal B. Lung and C. Breast tumour cohorts. Table S1. List of mutations analysed using the Agena MassArray technology. Table S2. Somatic mutations tested subdivided by pathway. Table S4. Antibodies used for Reverse Phase Protein Array (RPPA), including the company from which it was purchased, the catalog number, the host species and the dilution at which it was used. Table S6. Somatic mutation status in samples taken from two different regions of the same primary tumour. Samples identified in one sample but not in the other are identified in bold print. Table S7. Somatic mutation status of primary and matched metastatic tumour samples. Samples identified in one sample but not in the other are identified in bold print. Table S8. Frequency and co-occurrence of somatic PIK3CA and KRAS mutations in solid tumour samples. [file 12967_2020_2273_MOESM1_ESM.docx]

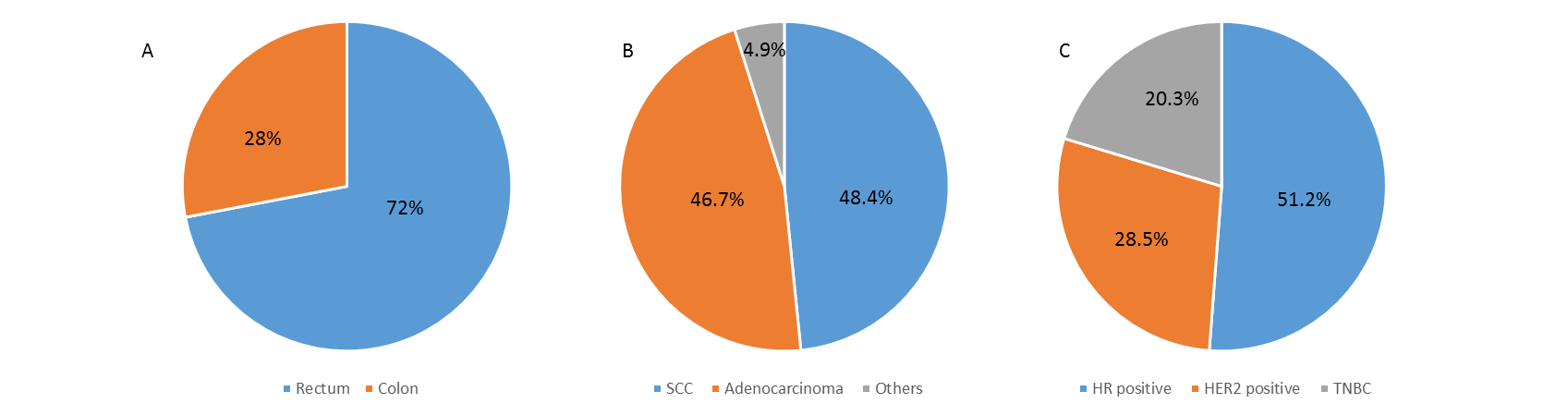


**Additional Figure S1. Breakdown of tumour types in A. Colorectal B. Lung and C. Breast tumour cohorts.** SCC; Squamous cell carcinoma, HR positive; Hormone Receptor Positive, HER2 positive, human epidermal growth factor receptor 2 receptor positive, TNBC; Triple Negative breast cancer. Others in lung cohort include large cell (n=5), adenosquamous (n=4), Bronchioloalveolar carcinoma (n=1) and carcinoid (n=1).

Additional Table S1: List of mutations analysed using the Agena MassArray technology

| **AKT1** | E17K, E49K, G173R, K179M |
| --- | --- |
| **AKT2** | E17K, G175R |
| **AKT3** | E17K, G171R |
| **ALK** | L560F, A877S, D1091N, M1166R, I1171N, F1174C/S/L/L/I/V, F1245C/L/V/I, R1275Q/L |
| **APC** | R1114X, E1306X, E1338X, Q1367X, E1379X, Q1429X, R1450X |
| **BRAF** | R444Q, R462I, I463S, G464E/V/A/R, G466R/E/V/A, G469A/E/R/V, V471F, Y472S, E586K, D587A/E, I592M/V, D594E/V/G, F595L/L/L/S, G596R, L597R/R/Q/V, T599I, V600E/A/G/L/M, K601E/N/N, S605N, G615R |
| **CDK4** | R24C/H |
| **CDKN1B** | P117S |
| **CDKN2A** | R58X, E61X, E69X, R80X, H83Y, E88X |
| **CTNNB1** | A13T, A21T, V22A, D32A/G/V/H/N/Y, S33A/P/T, G34E/V/A/R/R, I35N/S/T, H36P/R/Y, S37A/P/T/C/F/Y, T41A/P/S/I/N/S, S45A/P/T/C/F/Y |
| **DDR2** | R105S, N456S, T533K |
| **EGFR** | V689M, N700D, E709A/V/G/K/Q, G719A/D/C/S/R, S720T/P, D761N/Y, V769L/M, T783A, A839T, K846R, L858M/R, L861Q/R, G863D, H870R, E844K |
| **ERBB2** | S310F/Y, L755S, G776S/V, D769H, V777A/L/M, V842I, H878Y |
| **FBXO4** | S8R, S12L, L23Q, P76T |
| **FBXW7** | R465C/H/L, R479G/Q/L, R505C/S/H/L/P, S582L |
| **FGFR1** | S125L, P252T |
| **FGFR2** | S252W, Y375C, N549K/K |
| **FGFR3** | R248C, S249C, G370C, S371C, Y373C, G380R, A391E, K650E/Q/M/T, G697C |
| **GNA11** | Q209L/P, R138C |
| **GNAS** | R201H/S/C, Q227H/L/R |
| **GNAQ** | Q209L/P/R |
| **HRAS** | G12S/R/C/D/A/V, G13S/R/C, Q61H/H/Q/K/L/P/R, E62G |
| **IDH1** | G70D, R132C/G/S/H/L, V178I |
| **IDH2** | R172G/W/M/K/S |
| **KIT** | M552L, Y553N, W557G/R/R, K558N/R, V559A/D/G/I, V560D/A, G565R, N566D, Y568D, V569G, P573L, F584S, L576P, E561K, K642E, V654A, T670I, D716N, D816E/H/N/Y/G/V/A, D820E/E/H/Y/A/G, N822K/N/K/Y/H, Y823D/N |
| **KRAS** | G12D/A/V/S/R/C, KRASG13D/A/V/S/R/C, L19F/F, Q22K, T58I, A59T/G/E, G60D, Q61E/K/X/H/H/Q/L/P/R, A146P/T |
| **MAP2K1** | F53C/S, Q56P, K57N, P124L/T/S, E203K/Q |
| **MAP2K2** | E207K/Q, R388Q |
| **MAP3K13** | P373S, S694L, R880C, A882S |
| **MET** | E168D, N375S, R970C, T1010I, R1112R/L/Y, H1124D, M1131T, Y1248C/H/D, Y1253D, M1268T |
| **MLH1** | V384D |
| **MYC** | P57S, T58A |
| **NCOR1** | R108X, Q313X, E379X, I1422S, Q1792X |
| **NRAS** | G12D/A/V/S/R/C, G13D/A/V/S/R/C, A18T, Q61E/K/X/H/H/Q/R/P/L |
| **PDGFRA** | V561D, N659K/Y, D842Y/N/V, D846Y, Y849C, D1071N |
| **PIK3CA** | R38H, Q60K, R88Q, K111N, G118D, N345K, S405F, E418K, C420R, E453K, P539R, E542K/Q/V/G, E545D/K/Q/A/V/G, Q546H/E/K/L/P/R, C901F, F909L/L, M1004I, G1007R, Y1021C/H/N, R1023Q, T1025A/S/I, A1035T/V, M1043I/I/I/V, A1046V, H1047R/L/Y, G1049R, I1058F, H1065L, |
| **PIK3R1** | G376R, D560Y, N564D |
| **PTEN** | R130L/P/Q/X, R173C/H, R233X, R335X |
| **PTPN11** | S72D/V/T, E69K, E76A/G/V/Q/K |
| **RB1** | E137X, L199X, R320X, R358X, R455X, R552X, R556X, R579X, C706F, E748X |
| **RET** | C634R/W/Y, A664D, E768D, M918T |
| **SMARCD** | Q539X, D391H, Q504X |
| **SOS1** | R248H, R688Q, H888Q |
| **SRC** | Q531X |
| **STK11** | Q37X, Q170X, D194N/Y/V, G196V, E199X/K, P281L, W332X, F354L |
| **TBX3** | Y163X, W197X |
| **VHL** | P81X, L85P, L89H, L158Q/V, R161X, R167W |

Additional Table S2: Somatic mutations tested subdivided by pathway

| **Pathway** | **Genes** |
| --- | --- |
| Cell Cycle | CDK4, CDKN2A, GNA11, GNAQ, GNAS, MYC, RB1 |
| DNA damage response | FBXW7 |
| MAPK pathway | BRAF, HRAS, KRAS, NRAS, MAP2K1, MAP2K2, MAP3K13, PTPN11 |
| Metabolic | IDH1 |
| PI3K pathway | SKT, PIK3CA, PTEN, STK11 |
| RTK signalling | ERBB2, FGFR1, FGFR2, FGFR3, KIT, NCOR1 |
| Transcription regulation | TBX3 |
| Tumour suppressor genes | APC |
| Wnt/ β-catenin | CTNNB1 |

Additional table S4: Antibodies used for Reverse Phase Protein Array (RPPA), including the company from which it was purchased, the catalog number, the host species and the dilution at which it was used.

| **Antibody** | **Cat no** | **Company** | **Dilution** | **Host** |
| --- | --- | --- | --- | --- |
| AKT | 4691 | Cell Signalling | 1:3000 | Rabbit |
| AKT (S473) | 9271 | Cell Signalling | 1:250 | Rabbit |
| AKT (T308) | 2965 | Cell Signalling | 1:500 | Rabbit |
| AKT2 | 2964 | Cell Signalling | 1:50 | Rabbit |
| MAPK-ERK 1/2 | 9102 | Cell Signalling | 1:200 | Rabbit |
| MAPK (T202/Y204) -ERK1/2 | 4377 | Cell Signalling | 1:1200 | Rabbit |
| MEK1 | 1235-1 | Epitomics | 1:1200 | Rabbit |
| MEK1/2 (S217/221) | 9154 | Cell Signalling | 1:1000 | Rabbit |
| mTOR | 2983 | Cell Signalling | 1:400 | Rabbit |
| mTOR (S2448) | 2971 | Cell Signalling | 1:100 | Rabbit |
| p38_MAPK | 9212 | Cell Signalling | 1:300 | Rabbit |
| p38 MAPK (T180/Y182) | 9211 | Cell Signalling | 1:250 | Rabbit |
| p70 S6 kinase | 1494-1 | Epitomics | 1:250 | Rabbit |
| p70 S6 kinase (T389) | 9205 | Cell Signalling | 1:250 | Rabbit |
| PDK1 | 3062 | Cell Signalling | 1:100 | Rabbit |
| PDK1 (S241) | 3061 | Cell Signalling | 1:100 | Rabbit |
| PI3K p110alpha | 4255 | Cell Signalling | 1:100 | Rabbit |
| PKCalpha | 05-154 | Merck Millipore | 1:2000 | Mouse |
| PKCalpha (S657) | 06-822 | Merck Millipore | 1:3000 | Mouse |
| PTEN | 9552 | Cell Signalling | 1:100 | Rabbit |
| S6 ribosomal protein (S235/236) | 4856 | Cell Signalling | 1:200 | Rabbit |
| S6 ribosomal protein (S240/244) | 2215 | Cell Signalling | 1:3000 | Rabbit |

AKT, protein kinase B; MAPK, mitogen-activated protein kinase; MEK1, mitogen-activated protein kinase kinase; mTOR, mammalian target of rapamycin; p70 S6 kinase, ribosomal protein S6 kinase beta-1.

Additional Table S6: Somatic mutation status in samples taken from two different regions of the same primary tumour. Samples identified in one sample but not in the other are identified in **bold** print.

| **Sample number** | **Tumour Type** | **Sample 1 mutation status** | **Sample 2 mutation status** |
| --- | --- | --- | --- |
| 005 | Breast | PIK3CA E545K | PIK3CA E545K |
| 022 | Breast | WT | WT |
| 030 | Breast | HRAS G12R | HRAS G12R, **STK11 F354L** |
| 033 | Breast | WT | WT |
| 034 | Breast | PIK3CA C420R | PIK3CA C420R |
| 037 | Breast | STK11 F354L, PIK3CA H1047R | STK11 F354L, PIK3CA H1047R |
| 044 | Breast | PIK3CA E542K | PIK3CA E542K |
| 048 | Breast | WT | WT |
| 080 | Breast | PIK3CA E545K | PIK3CA E545K |
| 201 | Colorectal | APC E1379X, PTPN11 A72D | APC E1379X, PTPN11 A72D |
| 205 | Colorectal | KRAS G12D | KRAS G12D |
| 208 | Colorectal | KRAS G12D, PIK3CA H1047R, GNAS R201S | KRAS G12D, PIK3CA H1047R, GNAS R201S |
| 210 | Colorectal | BRAF V600E, APC Q1429X | BRAF V600E, APC Q1429X |
| 214 | Colorectal | KRAS G12D | KRAS G12D |
| 223 | Colorectal | GNAS R201C, BRAF V600E | GNAS R201C, BRAF V600E, **FBXW7 R505S** |
| 285 | Colorectal | WT | WT |
| 302 | Colorectal | KRAS G12V, APC Q1429X | KRAS G12V, APC Q1429X |
| 324 | Colorectal | WT | WT |
| 529 | Lung | KRAS G12V | KRAS G12V |
| 531 | Lung | PTEN R173H | PTEN R173H |
| 547 | Lung | WT | WT |
| 551 | Lung | KRAS G12D, PTPN11 E76A, PIK3CA E545K | KRAS G12D, PTPN11 E76A, PIK3CA E545K |
| 672 | Lung | KRAS G12C | KRAS G12C |
| 758 | Prostate | WT | WT |
| 767 | Prostate | WT | WT |
| 774 | Prostate | CTNNB1 D32G | CTNNB1 D32G |
| 781 | Prostate | PIK3CA E542K | PIK3CA E542K |
| 816 | Prostate | NCOR1 E379X | NCOR1 E379X |
| 841 | Melanoma | BRAF V600E | BRAF V600E |
| 844 | Melanoma | BRAF V600E | BRAF V600E |
| 852 | Melanoma | NRAS G12C | NRAS G12C |
| 953 | Gastric | NRAS G12C | NRAS G12C |
| 954 | Gastric | PIK3CA E442K, PTPN11 A72D | PIK3CA E442K, PTPN11 A72D |
| 957 | Gastric | WT | WT |
| 958 | Gastric | PIK3CA H1047R | PIK3CA H1047R |
| 986 | Gastric | PIK3CA Y1021C, KRAS G13D | PIK3CA Y1021C, KRAS G13D |
| 989 | Gastric | PIK3CA E542K | PIK3CA E542K |
| 1045 | Bladder | CDKN2A R80X | CDKN2A R80X |
| 1048 | Bladder | PIK3CA E545K | PIK3CA E545K |
| 1064 | Bladder | PIK3CA E542K | PIK3CA E542K |
| 1111 | Renal | NRAS G12C | NRAS G12C |
| 1113 | Renal | WT | WT |
| 1138 | Ovary | WT | WT |
| 1139 | Ovary | CTNNB1 S37A, **PTPN11 E76A** | CTNNB1 S37A |
| 1150 | Ovary | KRAS G12V | KRAS G12V |
| 1155 | Ovary | PIK3CA H1047R | PIK3CA H1047R |
| 1260 | Liver | WT | WT |
| 1273 | Testis | KIT Y823D | KIT Y823D |
| 1275 | Testis | WT | WT |
| 1299 | Sarcoma | WT | WT |

Additional Table S7: Somatic mutation status of primary and matched metastatic tumour samples. Samples identified in one sample but not in the other are identified in **bold** print.

| **Sample number** | **Primary tumour** | **TNM stage** | **Primary tumour mutation status** | **Metastasis site** | **Metastasis mutation status** |
| --- | --- | --- | --- | --- | --- |
| 001 | HR pos BC | T3N1M0 | STK11 F354L | Brain | STK11 F354L |
| 004 | TNBC | T2N0M0 | WT | Lung | WT |
| 035 | TNBC | T2N1M0 | PIK3CA H1047R, **PTPN11 A72D** | Lung | PIK3CA H1047R |
| 077 | HR pos BC | T1N1M0 | WT | Breast | **ERBB2 V777L** |
| 082 | TNBC | T1N2M0 | WT | Breast | WT |
| 093 | TNBC | T2N0M0 | PIK3CA H1047R | Lung | PIK3CA H1047R |
| 103 | TNBC | T2N2M0 | WT | LN | WT |
| 109 | TNBC | T2N0M0 | WT | Breast | WT |
| 173 | Colon | T3N2M0 | KRAS G12D | Pelvis | KRAS G12D |
| 177 | Colon | T2N0M0 | NRAS Q61L, FBXW7 R465C | Liver | NRAS Q61L, FBXW7 R465C |
| 215 | Colon | T3N1M0 | WT | Stomach | WT |
| 220 | Rectum | T3N1M0 | WT | Peritoneum | WT |
| 241 | Colon | Unknown | KRAS G13D, AKT1 E17K | LN | KRAS G13D, AKT1 E17K |
| 246 | Rectum | T2N0M0 | WT | Lung | **MAP3K13 S694L** |
| 247 | Rectum | T2N0M0 | KRAS G12S | Lung | KRAS G12S |
| 249 | Rectum | T2N0M0 | KRAS G12V, GNAS R201H | Liver | KRAS G12V, GNAS R201H |
| 253 | Rectum | Unknown | WT | LN | WT |
| 285 | Colon | T1N0M0 | KRAS G12D | Liver | KRAS G12D |
| 294 | Rectum | Unknown | CTNNB1 A21T | Colon | CTNNB1 A21T |
| 295 | Rectum | T3N1M0 | PIK3CA H1047R | Lung | PIK3CA H1047R |
| 835 | Melanoma | T3N1M0 | NRAS Q61R | Lung | NRAS Q61R |
| 844 | Melanoma | Unknown | BRAF V600E | Liver | BRAF V600E |
| 850 | Melanoma | T3N0M0 | KRAS Q61P | Right leg | KRAS Q61P |
| 884 | Melanoma | T3N2M0 | BRAF V600E | Right axilla | BRAF V600E |
| 950 | Gastric | T3N1M0 | WT | Liver | WT |
| 952 | Gastric | T3N2M0 | WT | Peritoneum | WT |
| 1042 | Bladder | T2N0M0 | FGFR3 R248C | LN | FGFR3 R248C |
| 1045 | Bladder | T3N0M0 | CDKN2A R80X | Liver | CDKN2A R80X |
| 1160 | Oesophagus | T3N1M0 | HRAS Q61H | Liver | HRAS Q61H |
| 1167 | Oesophagus | T2N0M0 | PIK3CA H1047Y | Abdominal wall | PIK3CA H1047Y |

HR pos BC, Hormone Receptor Positive breast cancer; TNBC, Triple Negative Breast Cancer; LN, Lymph node; WT, wildtype

Additional Table S8: Frequency and co-occurrence of somatic PIK3CA and KRAS mutations in solid tumour samples

| **Tumour Type** | **PIK3CA mutation (%)** | **KRAS Mutation (%)** | **Co-occurrence (%)** | | **Wildtype**  **(%)** |
| --- | --- | --- | --- | --- | --- |
| Colorectal | 8.2 | 35.3 | 6.2 | 50.3 | |
| Lung | 8.5 | 15.2 | 2.2 | 73.5 | |
| Breast | 29.7 | 0 | 0 | 70.3 | |
| Prostate | 4.8 | 0 | 0 | 95.2 | |
| Melanoma | 0 | 1.5 | 0 | 98.5 | |
| Lymphoma | 6.1 | 4.1 | 0 | 89.8 | |
| Gastric | 6.7 | 6.7 | 2.2 | 84.4 | |
| Head & Neck | 22.2 | 2.2 | 2.2 | 73.3 | |
| Bladder | 28.9 | 0 | 0 | 71.1 | |
| Ocular melanoma | 0 | 0 | 0 | 100 | |
| Endometrial | 13.8 | 3.4 | 3.4 | 79.3 | |
| Kidney | 3.6 | 0 | 0 | 96.4 | |
| Ovary | 12.5 | 20.8 | 0 | 66.7 | |
| Brain | 12.5 | 0 | 0 | 87.5 | |
| Oesophagus | 13.6 | 0 | 0 | 86.4 | |
| Pancreas | 0 | 63.2 | 0 | 36.8 | |
| Liver | 0 | 0 | 0 | 100 | |
| Testis | 6.3 | 0 | 0 | 93.7 | |
| Thyroid | 0 | 0 | 0 | 100 | |
| Sarcoma | 0 | 0 | 0 | 100 | |
