# Supplementary material for: Identification and clinical impact of potentially actionable somatic oncogenic mutations in solid tumor samples
Source: J Transl Med. 2020 Feb 22;18:99. doi: 10.1186/s12967-020-02273-4 (PMC7036178; doi:10.1186/s12967-020-02273-4)
Supplement: Supplementary file 2 — Additional file 2: Table S3. Cancer Genome Interpreter mutation analysis of the mutations identified in solid tumour samples analysed in our study. [file 12967_2020_2273_MOESM2_ESM.docx]

Additional Table S3: Cancer Genome Interpreter mutation analysis of the mutations identified in solid tumour samples analysed in our study.

| **Gene** | **Protein** | **cDNA** | **Driver** | **Driver_mut_prediction** | **Exon** | **gDNA** | **Gene_role** |
| --- | --- | --- | --- | --- | --- | --- | --- |
| GNAQ | p.Q209P | c.626A>C | known | TIER 1 | 5 | chr9:g.80409488T>G | Act |
| GNAQ | p.Q209L | c.626A>T | known | TIER 1 | 5 | chr9:g.80409488T>A | Act |
| CDKN2A | p.H83Y | c.247C>T | known | TIER 1 | 2 | chr9:g.21971111G>A | LoF |
| FGFR1 | p.P283T | c.847C>A | known | TIER 1 | 8 | chr8:g.38282209G>T | Act |
| MYC | p.P57S | c.169C>T | known | TIER 2 | 2 | chr8:g.128750632C>T | Act |
| BRAF | p.G464V | c.1391G>T | known | TIER 1 | 11 | chr7:g.140481417C>A | Act |
| BRAF | p.G466R | c.1396G>A | known | TIER 1 | 11 | chr7:g.140481412C>T | Act |
| BRAF | p.G466A | c.1397G>C | known | TIER 1 | 11 | chr7:g.140481411C>G | Act |
| BRAF | p.G469R | c.1405G>A | known | TIER 1 | 11 | chr7:g.140481403C>T | Act |
| BRAF | p.G469V | c.1406G>T | known | TIER 1 | 11 | chr7:g.140481402C>A | Act |
| BRAF | p.D594G | c.1781A>G | known | TIER 1 | 15 | chr7:g.140453154T>C | Act |
| BRAF | p.V600E | c.1799T>A | known | TIER 1 | 15 | chr7:g.140453136A>T | Act |
| BRAF | p.V600A | c.1799T>C | known | TIER 1 | 15 | chr7:g.140453136A>G | Act |
| BRAF | p.K601E | c.1801A>G | known | TIER 1 | 15 | chr7:g.140453134T>C | Act |
| KIT | p.Y823D | c.2467T>G | known | TIER 1 | 17 | chr4:g.55599341T>G | Act |
| KIT | p.N822K | c.2466T>G | known | TIER 1 | 17 | chr4:g.55599340T>G | Act |
| KIT | p.D820Y | c.2458G>T | known | TIER 1 | 17 | chr4:g.55599332G>T | Act |
| KIT | p.W557R | c.1669T>A | known | TIER 1 | 11 | chr4:g.55593603T>A | Act |
| FGFR3 | p.R248C | c.742C>T | known | TIER 1 | 7 | chr4:g.1803564C>T | Act |
| CTNNB1 | p.S45Y | c.134C>A | known | TIER 1 | 3 | chr3:g.41266137C>A | Act |
| CTNNB1 | p.S37F | c.110C>T | known | TIER 1 | 3 | chr3:g.41266113C>T | Act |
| CTNNB1 | p.S37A | c.109T>G | known | TIER 1 | 3 | chr3:g.41266112T>G | Act |
| CTNNB1 | p.H36Y | c.106C>T | known | TIER 1 | 3 | chr3:g.41266109C>T | Act |
| CTNNB1 | p.G34V | c.101G>T | known | TIER 1 | 3 | chr3:g.41266104G>T | Act |
| CTNNB1 | p.G34E | c.101G>A | known | TIER 1 | 3 | chr3:g.41266104G>A | Act |
| CTNNB1 | p.D32G | c.95A>G | known | TIER 1 | 3 | chr3:g.41266098A>G | Act |
| CTNNB1 | p.D32H | c.94G>C | known | TIER 1 | 3 | chr3:g.41266097G>C | Act |
| CTNNB1 | p.D32N | c.94G>A | known | TIER 1 | 3 | chr3:g.41266097G>A | Act |
| CTNNB1 | p.A21T | c.61G>A | known | passenger | 3 | chr3:g.41266064G>A | Act |
| PIK3CA | p.G1049R | c.3145G>C | known | TIER 1 | 21 | chr3:g.178952090G>C | Act |
| PIK3CA | p.H1047L | c.3140A>T | known | TIER 1 | 21 | chr3:g.178952085A>T | Act |
| PIK3CA | p.H1047R | c.3140A>G | known | TIER 1 | 21 | chr3:g.178952085A>G | Act |
| PIK3CA | p.H1047Y | c.3139C>T | known | TIER 1 | 21 | chr3:g.178952084C>T | Act |
| PIK3CA | p.M1043I | c.3129G>C | known | TIER 1 | 21 | chr3:g.178952074G>C | Act |
| PIK3CA | p.Y1021C | c.3062A>G | known | TIER 1 | 21 | chr3:g.178952007A>G | Act |
| PIK3CA | p.Q546E | c.1636C>G | known | TIER 1 | 10 | chr3:g.178936094C>G | Act |
| PIK3CA | p.Q546K | c.1636C>A | known | TIER 1 | 10 | chr3:g.178936094C>A | Act |
| PIK3CA | p.E545G | c.1634A>G | known | TIER 1 | 10 | chr3:g.178936092A>G | Act |
| PIK3CA | p.E545K | c.1633G>A | known | TIER 1 | 10 | chr3:g.178936091G>A | Act |
| PIK3CA | p.E542K | c.1624G>A | known | TIER 1 | 10 | chr3:g.178936082G>A | Act |
| PIK3CA | p.C420R | c.1258T>C | known | TIER 1 | 8 | chr3:g.178927980T>C | Act |
| PIK3CA | p.N345K | c.1035T>G | known | TIER 1 | 5 | chr3:g.178921553T>G | Act |
| PIK3CA | p.R88Q | c.263G>A | known | TIER 1 | 2 | chr3:g.178916876G>A | Act |
| IDH1 | p.R132H | c.395G>A | known | TIER 1 | 4 | chr2:g.209113112C>T | Act |
| GNAS | p.R201H | c.602G>A | known | TIER 1 | 8 | chr20:g.57484421G>A | Act |
| GNAS | p.R201C | c.601C>T | known | TIER 1 | 8 | chr20:g.57484420C>T | Act |
| NRAS | p.G12S | c.34G>A | known | TIER 1 | 2 | chr1:g.115258748C>T | Act |
| NRAS | p.G12C | c.34G>T | known | TIER 1 | 2 | chr1:g.115258748C>A | Act |
| NRAS | p.G12D | c.35G>A | known | TIER 1 | 2 | chr1:g.115258747C>T | Act |
| NRAS | p.G13R | c.37G>C | known | TIER 1 | 2 | chr1:g.115258745C>G | Act |
| NRAS | p.G13V | c.38G>T | known | TIER 1 | 2 | chr1:g.115258744C>A | Act |
| NRAS | p.Q61K | c.181C>A | known | TIER 1 | 3 | chr1:g.115256530G>T | Act |
| NRAS | p.Q61E | c.181C>G | known | TIER 1 | 3 | chr1:g.115256530G>C | Act |
| NRAS | p.Q61R | c.182A>G | known | TIER 1 | 3 | chr1:g.115256529T>C | Act |
| NRAS | p.Q61L | c.182A>T | known | TIER 1 | 3 | chr1:g.115256529T>A | Act |
| NRAS | p.Q61H | c.183A>T | known | TIER 1 | 3 | chr1:g.115256528T>A | Act |
| GNA11 | p.Q209L | c.626A>T | known | TIER 1 | 5 | chr19:g.3118942A>T | Act |
| STK11 | p.P281L | c.842C>T | known | passenger | 6 | chr19:g.1221319C>T | LoF |
| STK11 | p.E199K | c.595G>A | known | TIER 1 | 4 | chr19:g.1220502G>A | LoF |
| STK11 | p.D194V | c.581A>T | known | TIER 1 | 4 | chr19:g.1220488A>T | LoF |
| ERBB2 | p.V777L | c.2329G>C | known | TIER 1 | 20 | chr17:g.37881000G>C | Act |
| ERBB2 | p.G776S | c.2326G>A | known | TIER 1 | 20 | chr17:g.37880997G>A | Act |
| ERBB2 | p.D769H | c.2305G>C | known | TIER 1 | 19 | chr17:g.37880261G>C | Act |
| ERBB2 | p.L755S | c.2264T>C | known | TIER 1 | 19 | chr17:g.37880220T>C | Act |
| MAP2K1 | p.P124L | c.371C>T | known | TIER 1 | 3 | chr15:g.66729163C>T | Act |
| AKT1 | p.E17K | c.49G>A | known | TIER 1 | 2 | chr14:g.105246551C>T | Act |
| RB1 | p.C706F | c.2117G>T | known | TIER 1 | 21 | chr13:g.49037877G>T | LoF |
| CDK4 | p.R24C | c.70C>T | known | TIER 1 | 2 | chr12:g.58145431G>A | Act |
| KRAS | p.G12S | c.34G>A | known | TIER 1 | 2 | chr12:g.25398285C>T | Act |
| KRAS | p.G12R | c.34G>C | known | TIER 1 | 2 | chr12:g.25398285C>G | Act |
| KRAS | p.G12C | c.34G>T | known | TIER 1 | 2 | chr12:g.25398285C>A | Act |
| KRAS | p.G12D | c.35G>A | known | TIER 1 | 2 | chr12:g.25398284C>T | Act |
| KRAS | p.G12A | c.35G>C | known | TIER 1 | 2 | chr12:g.25398284C>G | Act |
| KRAS | p.G12V | c.35G>T | known | TIER 1 | 2 | chr12:g.25398284C>A | Act |
| KRAS | p.G13S | c.37G>A | known | TIER 1 | 2 | chr12:g.25398282C>T | Act |
| KRAS | p.G13R | c.37G>C | known | TIER 1 | 2 | chr12:g.25398282C>G | Act |
| KRAS | p.G13D | c.38G>A | known | TIER 1 | 2 | chr12:g.25398281C>T | Act |
| KRAS | p.L19F | c.57G>T | known | TIER 1 | 2 | chr12:g.25398262C>A | Act |
| KRAS | p.Q22K | c.64C>A | known | TIER 1 | 2 | chr12:g.25398255G>T | Act |
| KRAS | p.Q61K | c.181C>A | known | TIER 1 | 3 | chr12:g.25380277G>T | Act |
| KRAS | p.Q61E | c.181C>G | known | TIER 1 | 3 | chr12:g.25380277G>C | Act |
| KRAS | p.Q61P | c.182A>C | known | TIER 1 | 3 | chr12:g.25380276T>G | Act |
| KRAS | p.Q61R | c.182A>G | known | TIER 1 | 3 | chr12:g.25380276T>C | Act |
| KRAS | p.Q61H | c.183A>T | known | TIER 1 | 3 | chr12:g.25380275T>A | Act |
| KRAS | p.A146T | c.436G>A | known | TIER 1 | 4 | chr12:g.25378562C>T | Act |
| HRAS | p.G12R | c.34G>C | known | TIER 1 | 2 | chr11:g.534289C>G | Act |
| HRAS | p.G13R | c.37G>C | known | TIER 1 | 2 | chr11:g.534286C>G | Act |
| HRAS | p.Q61K | c.181C>A | known | TIER 1 | 3 | chr11:g.533875G>T | Act |
| HRAS | p.Q61L | c.182A>T | known | TIER 1 | 3 | chr11:g.533874T>A | Act |
| PTEN | p.R173H | c.518G>A | known | TIER 1 | 6 | chr10:g.89711900G>A | LoF |
| PTEN | p.R173C | c.517C>T | known | TIER 1 | 6 | chr10:g.89711899C>T | LoF |
| PTEN | p.R130Q | c.389G>A | known | TIER 1 | 5 | chr10:g.89692905G>A | LoF |
| FGFR2 | p.N550K | c.1650T>G | known | TIER 1 | 12 | chr10:g.123258034A>C | Act |
| FGFR2 | p.N639K | c.1917T>G | known | TIER 2 | 14 | chr10:g.123247577A>C | Act |
| BRAF | p.G464A | c.1391G>C | predicted | TIER 1 | 11 | chr7:g.140481417C>G | Act |
| KIT | p.D816N | c.2446G>A | predicted | TIER 1 | 17 | chr4:g.55599320G>A | Act |
| KIT | p.V603A | c.1808T>C | predicted | TIER 1 | 12 | chr4:g.55594022T>C | Act |
| KIT | p.V603I | c.1807G>A | predicted | TIER 1 | 12 | chr4:g.55594021G>A | Act |
| KIT | p.W557G | c.1669T>G | predicted | TIER 1 | 11 | chr4:g.55593603T>G | Act |
| FBXW7 | p.R465C | c.1393C>T | predicted | TIER 1 | 9 | chr4:g.153249385G>A | LoF |
| FBXW7 | p.R465H | c.1394G>A | predicted | TIER 1 | 9 | chr4:g.153249384C>T | LoF |
| FBXW7 | p.R465L | c.1394G>T | predicted | TIER 1 | 9 | chr4:g.153249384C>A | LoF |
| FBXW7 | p.R479G | c.1435C>G | predicted | TIER 1 | 10 | chr4:g.153247367G>C | LoF |
| FBXW7 | p.R479Q | c.1436G>A | predicted | TIER 1 | 10 | chr4:g.153247366C>T | LoF |
| FBXW7 | p.R505S | c.1513C>A | predicted | TIER 1 | 10 | chr4:g.153247289G>T | LoF |
| FBXW7 | p.R505H | c.1514G>A | predicted | TIER 1 | 10 | chr4:g.153247288C>T | LoF |
| CTNNB1 | p.T41A | c.121A>G | predicted | TIER 1 | 3 | chr3:g.41266124A>G | Act |
| CTNNB1 | p.I35S | c.104T>G | predicted | TIER 1 | 3 | chr3:g.41266107T>G | Act |
| CTNNB1 | p.G34A | c.101G>C | predicted | TIER 1 | 3 | chr3:g.41266104G>C | Act |
| MAP3K13 | p.S694L | c.2081C>T | predicted | TIER 1 | 11 | chr3:g.185191200C>T | ambiguous |
| MAP3K13 | p.P373S | c.1117C>T | predicted | TIER 1 | 6 | chr3:g.185167794C>T | ambiguous |
| PIK3CA | p.Y1021H | c.3061T>C | predicted | TIER 1 | 21 | chr3:g.178952006T>C | Act |
| PIK3CA | p.Y1021N | c.3061T>A | predicted | TIER 1 | 21 | chr3:g.178952006T>A | Act |
| PIK3CA | p.E453K | c.1357G>A | predicted | TIER 1 | 8 | chr3:g.178928079G>A | Act |
| PIK3CA | p.S405F | c.1214C>T | predicted | TIER 1 | 7 | chr3:g.178927451C>T | Act |
| PIK3CA | p.G118D | c.353G>A | predicted | TIER 1 | 3 | chr3:g.178917478G>A | Act |
| PIK3CA | p.R38H | c.113G>A | predicted | TIER 1 | 2 | chr3:g.178916726G>A | Act |
| GNAS | p.R201S | c.601C>A | predicted | TIER 1 | 8 | chr20:g.57484420C>A | Act |
| MAP2K2 | p.E207K | c.619G>A | predicted | TIER 1 | 6 | chr19:g.4101103C>T | Act |
| STK11 | p.D194Y | c.580G>T | predicted | TIER 1 | 4 | chr19:g.1220487G>T | LoF |
| KRAS | p.G10R | c.28G>A | predicted | TIER 1 | 2 | chr12:g.25398291C>T | Act |
| PTPN11 | p.E76G | c.227A>G | predicted | TIER 1 | 3 | chr12:g.112888211A>G | Act |
| PTPN11 | p.E76A | c.227A>C | predicted | TIER 1 | 3 | chr12:g.112888211A>C | Act |
| PTPN11 | p.A72D | c.215C>A | predicted | TIER 1 | 3 | chr12:g.112888199C>A | Act |
| HRAS | p.G12S | c.34G>A | predicted | TIER 1 | 2 | chr11:g.534289C>T | Act |
| FGFR2 | p.N638K | c.1914C>G | predicted | TIER 2 | 14 | chr10:g.123247580G>C | Act |
| PIK3CA | p.Q60K | c.178C>A | other | passenger | 2 | chr3:g.178916791C>A | Act |
| GNAS | p.Q227H | c.681G>T | other | passenger | 9 | chr20:g.57484597G>T | Act |
| BRAF | p.Y472S | c.1415A>C | other | passenger | 11 | chr7:g.140481393T>G | Act |
| AKT3 | p.E17K | c.49G>A | other | passenger | 3 | chr1:g.243859016C>T |  |
| PIK3CA | p.F909L | c.2725T>C | other | passenger | 19 | chr3:g.178947850T>C | Act |
| STK11 | p.F354L | c.1060T>C | other | passenger | 8 | chr19:g.1223123T>C | LoF |
