# Supplementary material for: Identification and clinical impact of potentially actionable somatic oncogenic mutations in solid tumor samples
Source: J Transl Med. 2020 Feb 22;18:99. doi: 10.1186/s12967-020-02273-4 (PMC7036178; doi:10.1186/s12967-020-02273-4)
Supplement: Supplementary file 3 — Additional file 3: Table S5. Percentage of somatic hotspot mutations in each tumour type. [file 12967_2020_2273_MOESM3_ESM.docx]

|  | **AKT** | **APC** | **BRAF** | **CDK4** | **CDKN2A** | **CTNNB1** | **ERBB2** | **FBXW7** | **FGFR1** | **FGFR2** | **FGFR3** | **GNA11** | **GNAS** | **GNAQ** | **HRAS** | **IDH1** | **KIT** | **KRAS** | **MAP2K1** | **MAP2K2** | **MAP3K13** | **MYC** | **NCOR1** | **NRAS** | **PIK3CA** | **PTEN** | **PTPN11** | **RB1** | **STK11** | **TBX3** |
| --- | --- | --- | --- | --- | --- | --- | --- | --- | --- | --- | --- | --- | --- | --- | --- | --- | --- | --- | --- | --- | --- | --- | --- | --- | --- | --- | --- | --- | --- | --- |
| Colorectal (n=354) | 1.4 | 12.4 | 6.8 | - | - | 3.4 | 0.7 | 8.5 | 6.7 | - | - | - | 2 | - | 0.7 | - | 0.6 | 41.5 | - | - | 1.3 | 0.7 | 0.7 | 3.4 | 14.7 | 1.3 | 4.6 | - | 5.6 | 0.7 |
| Lung (n=223) | - | - | 2.7 | - | 3.1 | 0.9 | - | 1.8 | 0.4 | - | - | - | - | - | 2.2 | - | 0.4 | 17.5 | 0.9 | - | - | 0.9 | - | 1.3 | 10.8 | 0.4 | 5.4 | 0.4 | 2.7 | 0.4 |
| Breast (n=172) | - | - | - | - | 0.6 | - | 1.2 | - | - | 0.6 | - | - | - | - | 0.6 | - | 0.6 | - | - | - | - | 1.2 | - | 1.2 | 30.2 | 1.7 | 3.5 | - | 1.2 | - |
| Prostate (n=83) | - | - | - | - | - | 1.2 | - | - | - | - | - | - | - | - | 1.2 | - | - | - | - | - | - | - | 1.2 | - | 4.8 | - | 1.2 | - | 1.2 | - |
| Melanoma (n=65) | - | - | 20 | - | - | 1.5 | - | - | - | - | - | - | - | - | - | - | 1.5 | 1.5 | 1.5 | 1.5 | - | 1.5 | - | 10.8 | - | - | - | - | - | - |
| Lymphoma (n=49) | - | - | - | - | - | - | - | - | - | - | - | - | - | - | 2 | - | - | 4.1 | - | - | 2 | - | - | 2 | 6.1 | - | 4.1 | - | - | - |
| Gastric (n=45) | - | - | 2.2 | - | - | - | - | - | - | - | - | - | - | - | - | - | 6.7 | 8.9 | - | - | - | - | - | 2.2 | 8.9 | - | 4.4 | - | 4.4 | - |
| Head & Neck (n=45) | - | - | 6.7 | - | 4.4 | 2.2 | - | 2.2 | - | - | - | - | - | - | 4.4 | - | - | 4.4 | - | - | - | - | - | 8.9 | 24.4 | - | 2.2 | - | 2.2 | - |
| Bladder (n=38) | - | - | - | - | 5.3 | - | - | - | - | - | 2.6 | - | - | - | - | - | - | - | - | - | - | - | - | - | 28.9 | - | 2.6 | - | - | - |
| Oc. Melanoma (n=32) | - | - | - | - | - | - | - | - | - | - | - | 53.1 | - | 37.5 | - | - | - | - | - | - | - | - | - | - | - | - | - | - | - | - |
| Endometrial (n=29) | - | - | 3.4 | - | - | 3.4 | 3.4 | - | - | 3.4 | - | - | - | - | - | - | - | 17.2 | - | - | - | - | - | 3.4 | 17.2 | 17.2 | 3.4 | 3.4 | - | - |
| Kidney (n=28) | - | - | - | - | - | - | - | - | - | - | - | - | - | - | - | - | - | - | - | - | - | 3.6 | - | 3.6 | 3.6 | - | - | - | 3.6 | - |
| Ovary (n=24) | - | - | - | - | - | 8.3 | - | 4.2 | - | - | - | - | - | - | - | - | - | 20.8 | - | - | - | - | - | 4.2 | 12.5 | - | 8.3 | - | - | - |
| Brain (n=24) | - | - | - | - | - | - | - | 8.3 | - | - | - | - | - | - | - | 8.3 | - | - | - | - | - | - | - | 4.2 | 12.5 | - | - | - | - | - |
| Oesophagus (n=22) | - | - | - | - | - | - | - | 4.5 | - | - | - | - | - | - | 4.5 | - | - | - | - | - | - | - | - | - | 13.6 | - | - | - | 4.5 | - |
| Pancreas (n=19) | - | - | - | - | 5.3 | 5.3 | - | - | - | - | - | - | - | - | - | - | - | 63.2 | - | - | - | - | - | - | - | - | - | - | - | - |
| Liver (n=16) | - | - | - | - | - | 12.5 | - | - | - | - | - | - | - | - | - | - | - | - | - | - | - | - | - | - | - | - | - | - | - | - |
| Testis (n=16) | - | - | - | - | - | - | - | - | - | - | - | - | - | - | - | - | 12.5 | - | - | - | - | - | - | - | 6.3 | - | - | - | - | - |
| Thyroid (n=13) | - | - | 7.7 | - | - | - | - | - | - | - | - | - | - | - | 7.7 | - | 7.7 | - | - | - | - | - | - | 7.7 | - | - | - | - | 7.7 | - |
| Sarcoma (n=3) | - | - | - | - | - | - | - | - | - | - | - | - | - | - | - | - | - | - | - | - | - | - | - | - | - | - | - | - | - | - |

Additional Table S5: Percentage of somatic hotspot mutations in each tumour type
